# Impact of low FODMAP sourdough bread on gut microbiota using an *in vitro* colonic fermentation model

**DOI:** 10.3389/fmicb.2024.1496022

**Published:** 2024-11-11

**Authors:** Fatma Koc, Elke Arendt, Aidan Coffey, R. Paul Ross, Catherine Stanton

**Affiliations:** ^1^APC Microbiome Ireland, Cork, Ireland; ^2^School of Microbiology, University College Cork, Cork, Ireland; ^3^Food Biosciences Department, Teagasc Moorepark Research Center, Fermoy, Ireland; ^4^School of Food and Nutritional Sciences, University College Cork, Cork, Ireland; ^5^Department of Biological Sciences, Munster Technological University, Cork, Ireland

**Keywords:** sourdough bread, low FODMAP, gut microbiota, fermentation, short chain fatty acid, 16S rRNA gene sequencing

## Abstract

This study explores the development of whole-grain sourdough bread with reduced FODMAP (fermentable oligosaccharides, disaccharides, monosaccharides, and polyols) content to offer dietary solutions for individuals with irritable bowel syndrome (IBS). Three sourdough breads were prepared using different lactic acid bacteria (LAB) strains including *Lactiplantibacillus plantarum* FST1.7 (SD-FST1.7), *Lacticaseibacillus paracasei* R3 (SD-R3), and *Pediococcus pentosaceus* RYE106 (SD-RYE106). A control sourdough bread was prepared using baker’s yeast (SD-control). *In vitro* digestion and *in vitro* colonic fermentation were employed on bread samples with cellulose (negative control) and inulin (positive control), followed by 16S rRNA sequencing and short-chain fatty acid (SCFA) analysis to evaluate the impact on gut microbiota and SCFA levels. Alpha and beta diversity did not reveal any significant differences within the groups following *in vitro* colonic fermentation (FDR > 0.05). Taxonomic analysis displayed Firmicutes as the predominant phylum across all fecal samples at the end of colonic fermentation. Actinobacteriota was significantly lower in cellulose fermented fecal samples compared to samples fermented with SD-Control (ANCOMBC, FDR = 0.02) and inulin (ANCOMBC, FDR = 0.0001). Fecal samples fermented with inulin had significantly higher Bacteroidota levels compared to those fermented with cellulose (ANCOMBC, FDR =0.002). Acetate levels were higher in fecal samples fermented with SD-FST1.7 compared to those fermented with SD-R3 and SD-RYE106 (*p* = 0.03 for both). Positive correlations between butyrate and *Lachnospira*, *Agathobacter*, and *Bifidobacterium* were observed, demonstrating the potential of sourdough fermentation to influence gut health and support IBS management.

## 1 Introduction

Cereal-based foods are pillars of nutrition, boasting a rich array of essential nutrients and are important components of a balanced diet (Mobley et al., 2013; Poutanen et al., 2022). Among these, whole-grain sourdough bread is distinguished by its heightened fiber content, biogenic compounds, vitamins, and minerals, contributing to a reduced risk of non-communicable diseases (Sakandar et al., 2019; Kissock et al., 2021; Lockyer and Spiro, 2020; Laskowski et al., 2019). However, individuals with irritable bowel syndrome (IBS) often face dietary restrictions due to the high concentrations of fermentable oligo-, di-, monosaccharides, and polyols (FODMAPs) in whole-grain products, including wheat bread (Whelan et al., 2011; Ispiryan et al., 2020; Koc et al., 2024). Consumption of FODMAPs can exacerbate gastrointestinal symptoms in IBS sufferers, influencing their quality of life and productivity (Corsetti et al., 2018; Bertin et al., 2024). The low-FODMAP diet has emerged as a cornerstone approach for managing IBS symptoms, characterized by the restriction of high-FODMAP foods and the promotion of low-FODMAP alternatives, effectively mitigating gastrointestinal distress among IBS sufferers (Gibson and Shepherd, 2010; Staudacher et al., 2011; Halmos et al., 2014; Bohn et al., 2015).

Despite the tangible relief offered by the low-FODMAP diet, it presents practical challenges, particularly in the realm of dietary diversity and accessibility (Staudacher et al., 2011; Bohn et al., 2015). Traditional bread varieties, including whole-grain sourdough bread, often contain high levels of FODMAPs, rendering them unsuitable for individuals with IBS. Furthermore, the limited availability of low-FODMAP bread options, primarily restricted to gluten-free variations, poses a barrier to dietary adherence and enjoyment (Ispiryan et al., 2022; Moroni et al., 2009; El Khoury et al., 2018).

Sourdough fermentation, a natural leavening process driven by lactic acid bacteria (LAB) and wild yeast, offers immense potential for FODMAP reduction in bread (Borowska et al., 2023; Menezes et al., 2021; Boakye et al., 2022). The fermentation process, characterized by the gradual breakdown of complex carbohydrates and proteins by microbial enzymes, facilitates the conversion of fermentable carbohydrates into simpler, more digestible forms (Siddiqui et al., 2023). This enzymatic activity, coupled with the acidification of the dough, creates an environment conducive to the degradation of FODMAPs, thereby potentially reducing their presence in the final bread product (Loponen and Ganzle, 2018).

In light of these challenges, our study aims to explore innovative strategies to develop whole-grain sourdough bread with significantly reduced FODMAP content, thus offering a viable solution for individuals managing IBS symptoms. Reducing FODMAPs in breads has been studied in recent years (Pejcz et al., 2023; Shewry et al., 2022). However, those studies focused on chemical properties of bread. Leveraging the essential capacity of sourdough fermentation to modulate FODMAP levels, we aimed to harness this traditional bread-making technique to produce gut-friendly bread options that align with the principles of the low-FODMAP diet. Our previous study aimed to select FODMAP-reducing LAB for the production of sourdough (Borowska et al., 2023). The selected LAB strains included *L. plantarum* FST1.7 (FST1.7), *L. paracasei* R3 (R3), and *P. pentosaceus* RYE106 (RYE106). These LAB strains, chosen for their ability to thrive in sourdough environments and ferment carbohydrates, served as the key drivers of FODMAP reduction in our bread formulation. In previous research by Borowska et al., these three strains were shown to reduce the FODMAPs including sorbitol, mannitol, fructans, oligosaccharides and polyols significantly in sourdough bread. The details of formulation and biochemical properties of each sourdough bread have been previously published by Borowska et al. (2023).

In this current study, through a comprehensive investigation employing *in vitro* digestion, *in vitro* colonic fermentation, 16S rRNA sequencing, and short-chain fatty acid (SCFA) analysis, we investigated the effects of these fortified breads on gut microbiota and metabolome changes. Our findings illuminated the potential mechanisms underlying the health-promoting effects of fortified sourdough breads, with significant implications for the development of functional foods aimed at enhancing gut health.

## 2 Materials and methods

### 2.1 Sourdough bread preparation

Sourdough breads were prepared in four different recipes as previously described (Borowska et al., 2023). Three sourdough breads were prepared using different LAB strains: *L. plantarum* FST1.7 (SD-FST1.7), *L. paracasei* R3 (SD-R3), and *P. pentosaceus* RYE106 (SD-RYE106). A control sourdough bread was prepared using baker’s yeast (SD-control).

### 2.2 *In vitro* digestion

Four types of sourdough bread using different LAB strains: *L. plantarum* FST1.7 (SD-FST1.7), *L. paracasei* R3 (SD-R3), and *P. pentosaceus* RYE106 (SD-RYE106), control sourdough bread with baker’s yeast (SD-control), along with cellulose (negative control) and inulin (positive control), underwent *in vitro* digestion prior to *in vitro* colonic fermentation. The *in vitro* oral, gastric, and intestinal digestion process followed the INFOGEST 2.0 method to replicate the oral, gastric, and intestinal phases, including both negative and positive controls (Egger et al., 2016; Minekus et al., 2014; Brodkorb et al., 2019). Simulated salivary, gastric, and intestinal fluids were prepared at a stock concentration of 1.25x for each respective phase. During the oral phase, *α*-amylase (1,500 U mL^−1^, Sigma, A0521) was added to the simulated salivary fluid electrolyte solution and incubated at 37°C with the food samples for 2 min. In the gastric phase, the liquid samples from the oral phase were mixed with simulated gastric fluid electrolyte solution and pepsin (25,000 U mL^−1^, Sigma, P6887), adjusted to pH 3.0, and incubated in an incubating rocking platform shaker (VWR, Radnor, PA, United States) at 37°C for 2 h. The intestinal phase included the gastric chyme, simulated intestinal fluid electrolyte solution, pancreatin (800 U mL^−1^, Sigma, P7545), and fresh bile (10 mM), which were adjusted to pH 7.0 and incubated in an incubating rocking platform shaker at 37°C for 2 h. Afterward, the post-intestinal phase liquids were transferred to dialysis tubes and immersed in water as the dialysate for 24 h. The water was refreshed at 1, 6, and 18 h, after which the retentate was removed from the dialysis tubes. Retentates were then transferred to sterile 110 mm × 110 mm petri dishes and freeze dried for 24 h according to a previously described method (Koc et al., 2022).

### 2.3 *In vitro* colonic fermentation

Subsequent to *in vitro* digestion, *in vitro* colonic fermentation was conducted based on a previously established protocol (Koc et al., 2022). Participant recruitment and stool sample collection were approved by the Clinical Research Ethics Committee of the Cork Teaching Hospitals [review reference numbers: ECM 4 (gg) 11/02/20 & ECM 3 (iiii) 22/02/2022]. Fecal samples were obtained from nine healthy individuals for use in a MicroMatrix™ *in vitro* benchtop fermentation system, adhering to a protocol outlined in previous literature (O'Donnell et al., 2016). For each micromatrix well, 0.25 g of pre-digested and freeze dried sample was mixed with 4 mL of fecal fermentation media and 1 mL of fecal slurry. *In vitro* colonic fermentation was conducted in triplicate for the negative control (cellulose) and positive control (inulin), while pre-digested bread samples were evaluated in six replicates. Fecal fermentation was performed for 12 h and samples were collected at the baseline (T0) and at the end of fermentation (T12). These samples were collected into 2 mL sterile screw cap tubes. Subsequently, the tubes were centrifuged at 16,000 *g* for 10 min at 4°C and the pellet was utilized for DNA extraction to analyze microbial composition, while the supernatant was used for SCFA analysis.

### 2.4 DNA extraction and 16S rRNA gene library preparation

DNA extraction was performed using repeated bead beading protocol which was developed by Yu and Morrison (2004) and described in detail by Koc et al. (2022). Pellets were resuspended using 1 mL of lysis buffer composed of 500 mM NaCl, 50 mM Tris–HCl (pH 8.0), 50 mM EDTA, and 4% sodium dodecyl sulfate (SDS). The resulting suspension was transferred to 2 mL screw-cap microtubes (Sarstedt, Nümbrecht, Germany) containing Zirconia/Silica beads (0.125 g each of 0.1 mm and 1.5 mm beads, along with a single 2.5 mm bead; Biospec, Oklahoma, United States). The mixture was subjected to mechanical disruption using a bead beater (Biospec, Oklahoma, United States) at 4,000 rpm for 3 min. Following this, the tubes were incubated at 70°C for 15 min. The samples were then centrifuged at 16,000 *g* for 5 min at 4°C. The supernatant was carefully transferred to a sterile 2 mL Eppendorf tube, while 300 μL of the lysis buffer was added to the pellet, and the homogenization, incubation, and centrifugation steps were repeated. The collected supernatants were combined in a 2 mL tube. Next, 260 μL of chilled 7.5 M ammonium acetate was added to the supernatant, and the mixture was incubated on ice for 5 min. The tubes were centrifuged again at 16,000 *g* for 10 min at 4°C, after which 650 μL of the supernatant was transferred into two separate 1.5 mL tubes. An equal volume (650 μL) of isopropanol was added to each tube, and the samples were incubated overnight at −20°C. After incubation, the samples were centrifuged at 16,000 *g* for 15 min at 4°C. The supernatant was discarded, and the pellet was washed with 200 μL of 70% ethanol. The pellet was then resuspended in 100 μL of Tris-EDTA buffer. To degrade RNA, 2 μL of RNase (10 mg/mL) was added, followed by incubation at 37°C for 15 min. Afterward, 15 μL of proteinase K (from the QIAamp DNA Stool Mini Kit) and 200 μL of AL buffer (from the same kit) were added to the samples, which were incubated at 70°C for 10 min. Subsequently, 200 μL of absolute ethanol was added to precipitate the DNA. The samples were then transferred to QIAamp mini columns, and the wash and elution steps were carried out according to the QIAamp kit protocol. Finally, the extracted DNA was stored at −20°C until it was needed for 16S rRNA gene library preparation.

DNA samples were processed following the Illumina 16S Metagenomics Sequencing Library Preparation protocol. Amplification targeted the V3-V4 regions of the 16S rRNA gene using 16S Amplicon PCR Forward Primer = 5′ TCGTCGGCAGCGTCAGATGTGTATAAGAGACAGCCTACGGGNGGCWGCAG and 16S Amplicon PCR Reverse Primer = 5′ GTCTCGTGGGCTCGGAGATGTGTATAAGAGACAGGACTACHVGGGTATCTAATC. PCR reactions were carried out on a 2,720 thermal cycler (Life Technologies) using KAPA HiFi HotStart ReadyMix. The PCR conditions consisted of an initial denaturation at 95°C for 5 min, followed by 25 cycles of 95°C for 30 s, 55°C for 30 s, and 72°C for 30 s, with a final elongation step at 72°C for 5 min. For indexing, the Illumina Index primer set was applied following the manufacturer’s instructions. The resulting PCR amplicons were visualized on a 1.5% agarose gel (Sigma-Merck, Germany) stained with SYBR Safe (Thermo Fisher, MA, United States) to confirm amplification. Subsequently, PCR products were purified using the AMPure XP bead system (Beckman Coulter, CA, United States) in accordance with the Illumina protocol. Prior to pooling, the concentration of the purified products was quantified using the Qubit dsDNA High-Sensitivity kit (Thermo Fisher, MA, United States) and the Qubit 3.0 Fluorometer (Thermo Fisher, MA, United States). Following library preparation, all subsequent libraries were precisely pooled to achieve a final concentration of 4 nM. Afterwards, sequencing was performed utilizing the NextSeq 2000 platform, leveraging the high-throughput capabilities of this technology. Specifically, sequencing utilized the P1 600-cycle (2 × 300 bp) reagent kit sourced from Illumina (San Diego, United States). The sequencing procedures were conducted at the Teagasc Moorepark Research Center Next Generation DNA Sequencing Facility.

### 2.5 Short chain fatty acid analysis

Samples were prepared for SCFA analysis according to a previously described protocol with minor adaptations as explained in the work by Lynch et al. (2021). The standards and samples underwent analysis via gas chromatography—flame ionization detection (GC-FID) employing an Agilent 8860 GC system equipped with a DB-FFAP column (30 m length, 0.32 mm internal diameter, 0.25 μM film thickness; Agilent, California, United States) coupled with a flame ionization detector. Automated sample loading via splitless injection was facilitated using an autosampler. Helium served as the carrier gas, maintaining a constant flow rate of 1.3 mL/min throughout the analysis. The initial temperature of the GC oven was set at 50°C and held for 0.5 min, followed by a programmed ramp to 140°C at a rate of 10°C/min, where it was maintained for an additional 0.5 min. Subsequently, the temperature was elevated to 240°C at a rate of 20°C/min and held for 5 min. The total run time encompassed 20 min. The detector and injection port temperatures were set at 300°C and 240°C, respectively.

Peak integration was accomplished using Agilent OpenLab (version 2.0) software. Concentrations of SCFAs were reported in milimolar (mM) units for each compound. To discriminate significant differences among experimental groups, a one-way ANOVA with Tukey’s Honestly Significant Difference (HSD) *post hoc* test was employed. Statistical analyses were conducted utilizing the ‘ggpbur ()’ package, with visualization facilitated through the ‘ggplot2 ()’ package in R version 4.3.2, ensuring robust statistical assessment and clear representation of data trends via box plots.

### 2.6 Bioinformatics and statistical analysis

The raw sequencing data underwent processing via the DADA2 pipeline (version 1.16) as outlined by Callahan et al. (2016), incorporating specific parameters for optimal data refinement. These parameters included maxN = 0, maxEE = c (2, 2), rm.phix = TRUE, truncQ = 2, trimLeft = c (17, 21), truncLen = c (280,260), and maxN = 0, ensuring stringent quality control and accurate sequence inference. Next, the processed data were utilized to generate an amplicon sequence variant (ASV) table and perform taxonomic assignment using SILVA v138.1 database (Quast et al., 2013). ASV table has been provided in Supplementary Table 1. Integration of sample and group metadata, ASV table, and taxonomic information facilitated the creation of a phyloseq object, enabling comprehensive downstream analyses (McMurdie and Holmes, 2013).

Alpha diversity metrics, including Observed, Chao1, Shannon, and Simpson indices were computed to characterize within-sample diversity. Beta diversity was assessed utilizing the Bray-Curtis distance matrix and visualized through principal coordinate analysis (PCoA) to elucidate between-sample dissimilarities.

Statistical analyses encompassed Dunn’s test to discern significant differences in alpha diversity measurements and taxonomic compositions at both phylum and genus levels. False discovery rate (FDR) was used to correct *p* values for multiple comparisons. FDR values were considered as significant if *q* < 0.05. Furthermore, to assess differential abundance of microbial taxa across the six experimental groups, we employed the Analysis of Compositions of Microbiomes with Bias Correction (ANCOM-BC). This method accounts for compositional data characteristics and applies bias correction to identify significant differences in microbial abundance (Lin and Peddada, 2020). Cellulose (negative control) was used as the reference group for pairwise comparisons with the remaining groups. All statistical analyses were performed and graphics were generated using R (version 4.3.2). The details of bioinformatics analysis have been reported in Supplementary methods.

## 3 Results

### 3.1 Alpha and beta diversity

A total of 20,857,343 raw reads (2 × 300 bp) were obtained after sequencing with an average of reads per sample 651,791. After filtering and merging forward and reverse reads, we obtained a total of 13,159,481 reads with an average value of 411,233 reads/sample and a sequence length of 300 bp.

**Figure 1:**
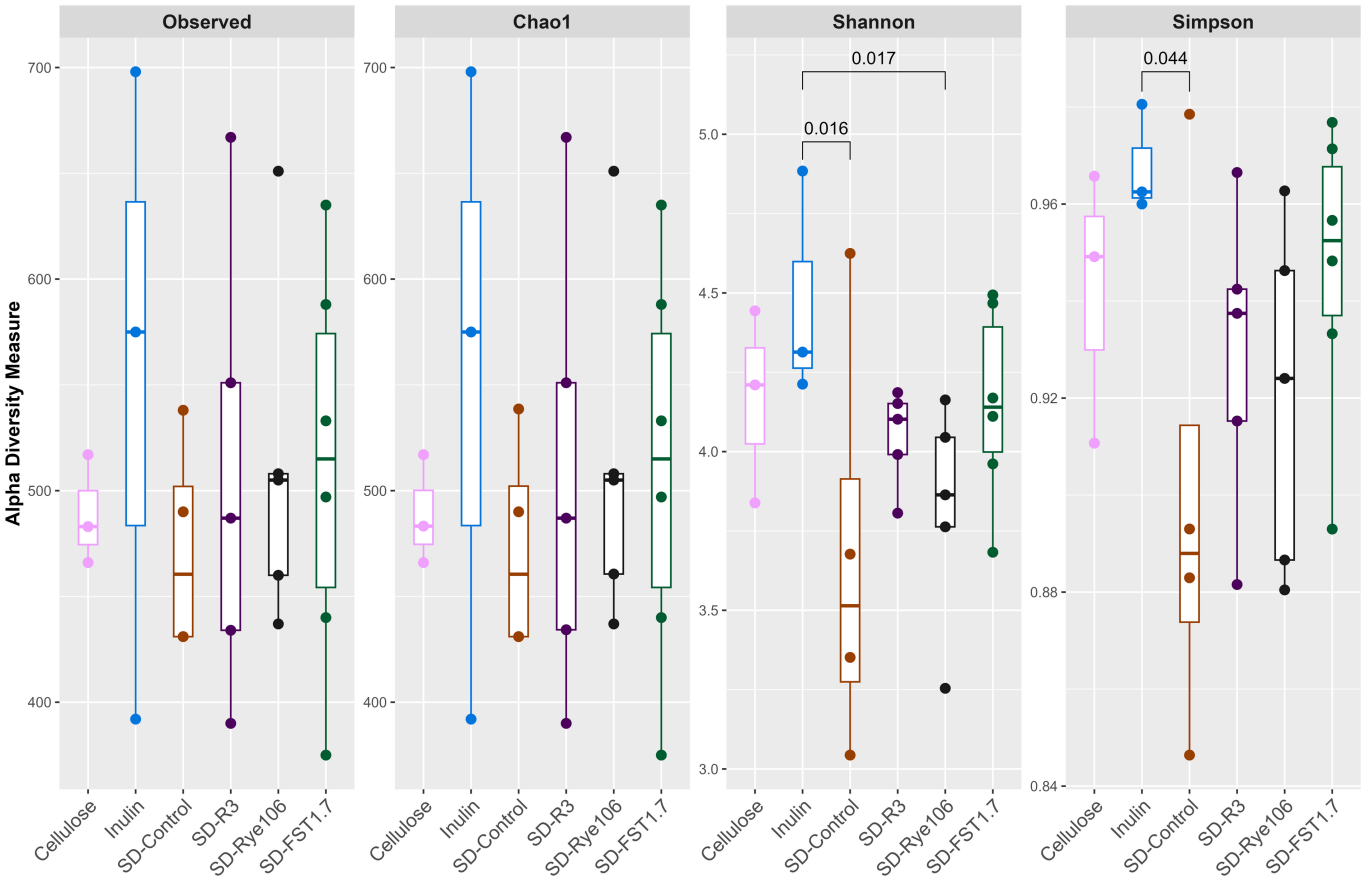
Alpha diversity assessed using Observed, Chao1, Shannon and Simpson indices following *in vitro* colonic fermentation of cellulose (negative control), inulin (positive control), sourdough bread fermented with baker’s yeast (SD-control), *Lacticaseibacillus paracasei* R3 (SD-R3), *Pediococcus pentosaceus* RYE106 (SD-Rye106), and *Lactiplantibacillus plantarum* FST1.7 (SD-FST1.7). Significance was determined using the Dunn’s test (FDR > 0.05 for all comparisons).

Alpha diversity was calculated (Figure 1) using four indices: Observed, Chao1, Shannon and Simpson following *in vitro* colonic fermentation of cellulose (negative control), inulin (positive control), sourdough bread fermented with baker’s yeast (SD-control), sourdough bread fermented with *L. paracasei* R3 (SD-R3), sourdough bread fermented with *P. pentosaceus* RYE106 (SD-Rye106) and sourdough bread fermented with *L. plantarum* FST1.7 (SD-FST1.7). Shannon diversity was found to be higher in the presence of inulin compared to SD-Control (*p* = 0.016, FDR = 0.24) and SD-Rye106 (*p* = 0.017, FDR = 0.24) at the end of colonic fermentation. Similarly, inulin had greater diversity in Simpson index compared to SD-control (*p* = 0.044, FDR = 0.66). Following false discovery rate (FDR) corrections, the comparisons were not significant (*q* > 0.05).

**Figure 2:**
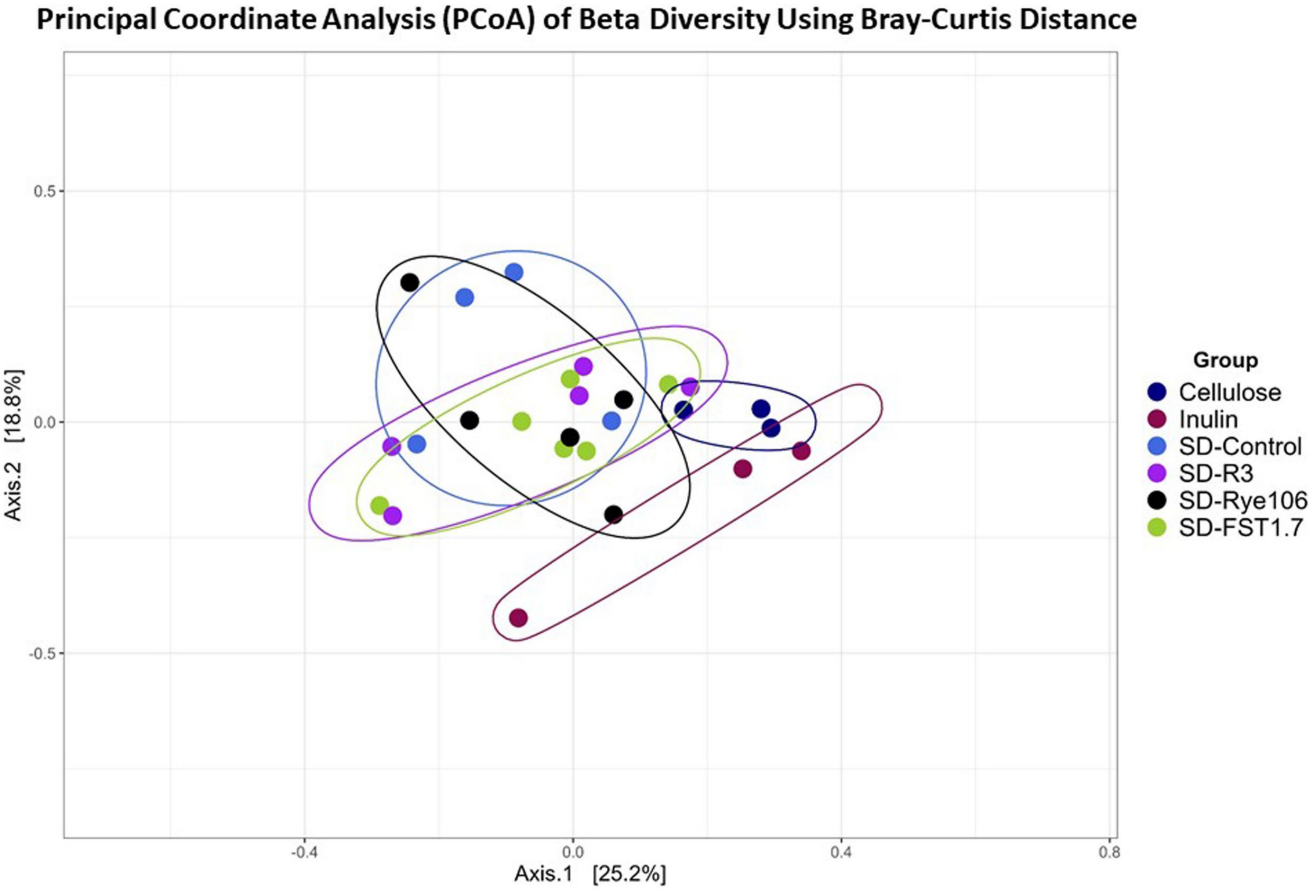
Principal Coordinate Analysis (PCoA) plot showing beta diversity of microbial communities following *in vitro* colonic fermentation of cellulose (negative control), inulin (positive control), SD-control, SD-R3, SD-Rye106, and SD-FST1.7.

Beta diversity was measured using Bray-Curtis distance metric and principal coordinate analysis plot (Figure 2). Table 1 shows the Pairwise PERMANOVA results with FDR value indicating no significant separation between the groups.

**Table 1:** Pairwise PERMANOVA results of each fermentation group following *in vitro* colonic fermentation.

Comparisons	Df	Sums of squares	*F*-Model	*R*^2^	*p* value	Adjusted *p* value (FDR)
SD-Rye106 vs. SD-FST1.7	1	0.069583388	0.642266446	0.06660949	0.819	0.819
SD-Rye106 vs. SD-R3	1	0.11814777	0.983747924	0.109503064	0.47	0.542
SD-Rye106 vs. Control	1	0.172949389	1.328100476	0.159472197	0.222	0.302
SD-Rye106 vs. Cellulose	1	0.400875202	3.831855028	0.389738764	0.019	0.052
SD-Rye106 vs. Inulin	1	0.371895062	2.433193306	0.288525736	0.019	0.052
SD-FST1.7 vs. SD-R3	1	0.069734891	0.772130407	0.079013519	0.526	0.563
SD-FST1.7 vs. Control	1	0.182910112	1.916295024	0.193247077	0.075	0.125
SD-FST1.7 vs. Cellulose	1	0.358277371	5.227753322	0.427531795	0.019	0.052
SD-FST1.7 vs. Inulin	1	0.338676131	3.082511232	0.305728519	0.006	0.052
SD-R3 vs. Control	1	0.136789842	1.277842143	0.154368991	0.276	0.345
SD-R3 vs. Cellulose	1	0.324895248	4.187966012	0.411069884	0.017	0.052
SD-R3 vs. Inulin	1	0.31383964	2.49466764	0.293674543	0.057	0.106
Control vs. Cellulose	1	0.374966384	4.504268494	0.473920586	0.021	0.052
Control vs. Inulin	1	0.374145315	2.651294499	0.346515808	0.055	0.106
Cellulose vs. Inulin	1	0.25388378	2.408039482	0.375784121	0.1	0.15

### 3.2 Gut microbial composition

**Figure 3:**
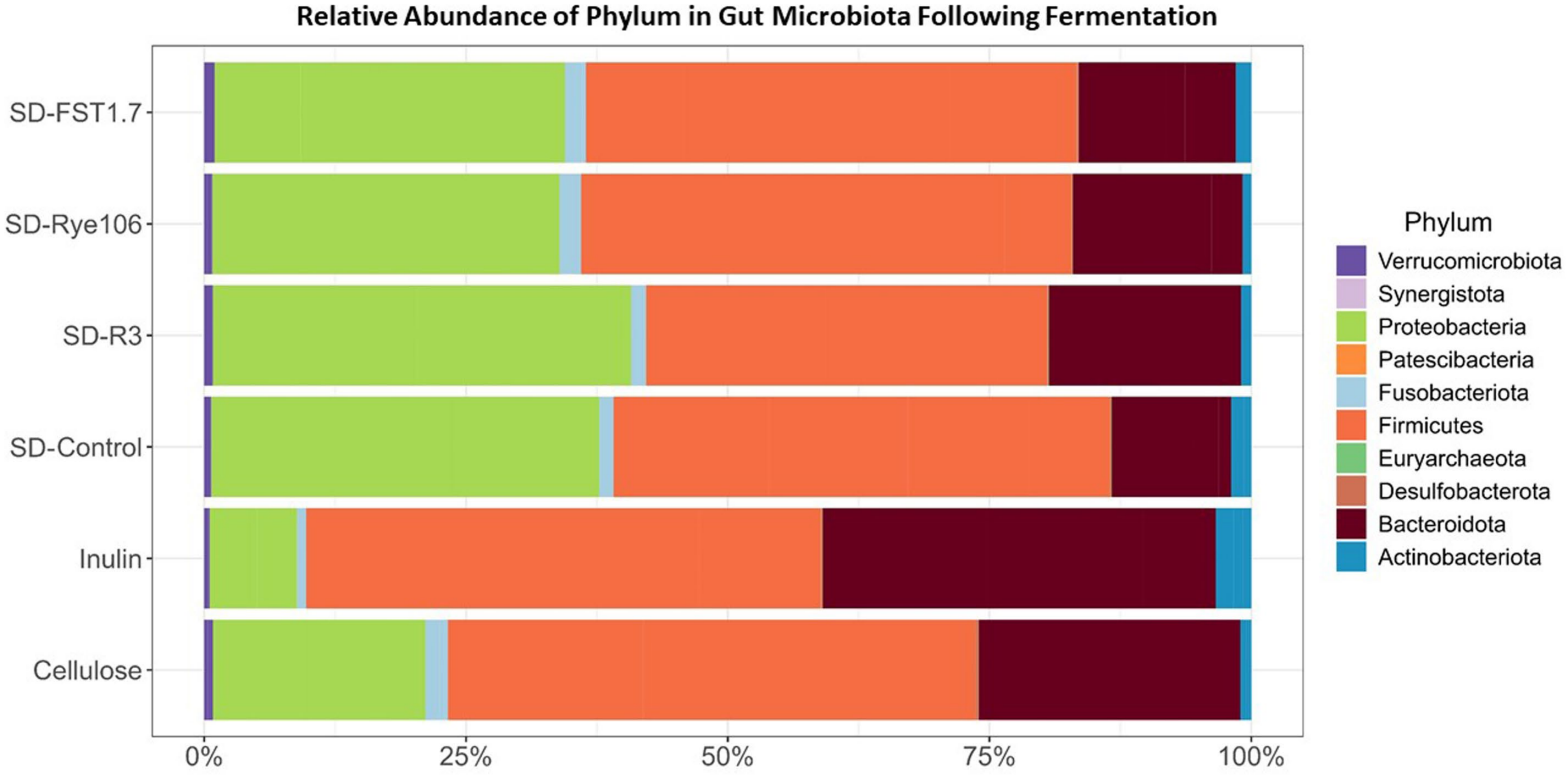
Bar charts showing taxonomic composition of gut microbiota at the phylum level (%) before (Baseline) and after *in vitro* colonic fermentation of cellulose (negative control), inulin (positive control), SD-control, SD-R3, SD-Rye106, and SD-FST1.7.

A total of nine phyla and 173 genera were identified, with Firmicutes being the dominant phylum across all samples, followed by Proteobacteria and Bacteroidota (Figure 3). Notably, no significant differences in Firmicutes abundance were observed between the groups. Statistical differences determined by Dunn’s test are provided in Supplementary Table 2. Moreover, the Proteobacteria levels exhibited significant variations, with higher relative abundances observed in fecal samples fermented with SD-R3 compared to fecal samples fermented with inulin (*p* = 0.00296, FDR = 0.044) following *in vitro* colonic fermentation. Actinobacteriota demonstrated a higher relative abundance in fecal samples containing inulin compared to those with SD-R3 and SD-Rye106 (*p* = 0.017 and *p* = 0.004) at the end of colonic fermentation, however after correcting *p* values, the differences were not significant (FDR = 0.13 and FDR = 0.06). Notably, fecal samples harboring SD-Control exhibited elevated Actinobacteriota levels relative to those containing SD-Rye106 (*p* = 0.033, FDR = 0.14). However, at the phylum level, a discrepancy was noted in the relative abundance of Bacteroidota, with higher levels detected in fecal samples fermented with inulin compared to those fermented with SD-FST1.7 (*p* = 0.033, FDR = 0.17). Furthermore, Desulfobacterota levels were elevated in fecal samples fermented with cellulose at T12 compared to fecal samples fermented with SD-R3 (*p* = 0.01, FDR = 0.07), SD-Rye106 (*p* = 0.01, FDR = 0.07), and SD-FST1.7 (*p* = 0.033, FDR = 0.12).

**Figure 4:**
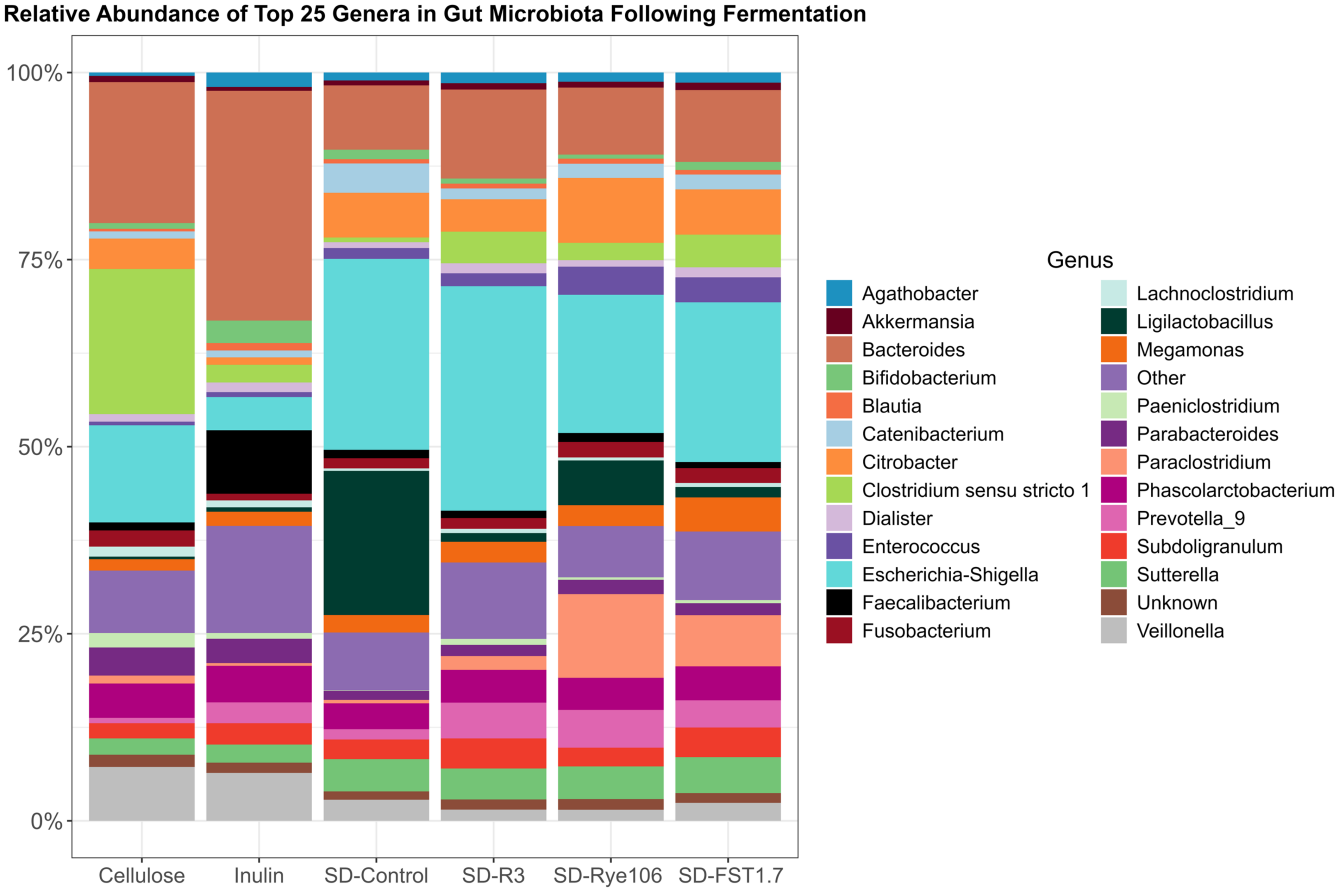
Bar charts depicting gut microbiota composition at the genus level across fermentation groups, including cellulose, inulin, SD-Control, SD-R3, SD-Rye106, and SD-FST1.7. The top 25 genera’s relative abundances (%) are displayed and the remainders are categorized in other.

The top 25 genera were used to construct a bar chart (Figure 4), illustrating gut microbiota alterations across the fecal samples after *in vitro* colonic fermentation with various substrates. Statistical differences between the groups were assessed using Dunn’s test, and the results are provided in Supplementary Table 3. *Agathobacter* exhibited higher levels in fecal samples fermented with SD-R3 and SD-Rye106 compared to samples fermented with cellulose (*p* = 0.01, FDR = 0.13 and *p* = 0.04, FDR = 0.18). Conversely, the relative abundance of *Bacteroides* was lower in the samples fermented with SD-FST1.7 compared to samples fermented with cellulose (*p* = 0.03, FDR = 0.089) and inulin (*p* = 0.03, FDR = 0.089). Similarly, fecal samples had lower levels of *Bacteroides* in the presence of SD-Rye106 compared to cellulose at the end of colonic fermentation (*p* = 0.031, FDR = 0.089). *Bifidobacterium* levels were significantly elevated in samples fermented with inulin compared to samples fermented with SD-Rye106 (*p* = 0.002, FDR = 0.04). In contrast, fecal samples exhibited significantly higher levels of C*itrobacter* in the presence of SD-Rye106 and SD-FST1.7 compared to those with inulin (*p* = 0.002, FDR = 0.035 and *p* = 0.006, FDR = 0.05). *Ligilactobacillus* levels were greater in fecal samples fermented with SD-Control compared to those fermented with cellulose (*p* = 0.004, FDR = 0.06). Fecal samples had lower *Veillonella* levels in the presence of SD-R3 and SD-Rye106 compared to cellulose (*p* = 0.001, FDR = 0.077 and *p* = 0.006, FDR = 0.077). *Clostridium sensu stricto 13* was found to be significantly higher in samples fermented with cellulose and inulin compared to those fermented with SD-Control (*p* = 0.003, FDR = 0.01 and *p* = 0.003, FDR = 0.01), SD-R3 (*p* = 0.001, FDR = 0.01 and *p* = 0.002, FDR = 0.01), SD-FST1.7 (*p* = 0.01, FDR = 0.03 and *p* = 0.01, FDR = 0.03). Similarly, *Clostridium sensu stricto 1* was significantly lower in samples fermented with SD-Control (*p* = 0.003, FDR = 0.04) and SD-Rye106 (*p* = 0.008, FDR = 0.05). The relative abundance of *Limosilactobacillus* was significantly greater in samples fermented with SD-FST1.7 and SD-control compared to those fermented with cellulose (*p* = 0.0004, FDR = 0.006, *p* = 0.005, FDR = 0.03). Similarly, SD-FST1.7 fermentates had higher levels of *Limosilactobacillus* compared to inulin fermentates (*p* = 0.006, FDR = 0.03).

The relative abundances of *Lactobacillus*, *Megamonas*, *Parabacteroides*, and *Prevotella*_9 were found to be similar across all fecal samples at the end of fermentation (*q* > 0.05).

### 3.3 Analysis of compositions of microbiomes with Bias correction

We conducted an Analysis of Compositions of Microbiomes with Bias Correction (ANCOM-BC) to evaluate differential microbial abundance across the six experimental groups, using cellulose (negative control) as the reference group for pairwise comparisons. The results, detailed in Supplementary Table 4, revealed significant log fold changes (lfc) in microbial taxa abundance at genus level with corresponding q values to indicate statistical significance (*q* < 0.05). All experimental groups demonstrated a significantly greater proportion of *Agathobacter* compared to cellulose (*q* < 0.05). In contrast, *Akkermansia* abundance was significantly lower in both the inulin and SD-R3 groups relative to cellulose (*q* < 0.05). *Bacteroides* levels were significantly reduced in SD-control, while *Catenibacterium* abundance was higher compared to SD-control (*q* < 0.05). In the SD-Rye106 group, *Citrobacter* abundance increased significantly compared to cellulose (lfc = 1.03, *q* < 0.05). *Clostridium sensu stricto 1* and *Clostridium sensu stricto 13* were reduced in all sourdough bread groups, with the exception of inulin (*q* < 0.05), while *Dialister* abundance increased 0.3-fold in SD-FST1.7 relative to cellulose (*q* < 0.05). *Enterococcus* levels were higher in SD-Rye106 and SD-FST1.7 compared to cellulose. The abundance of *Escherichia-Shigella* was significantly higher in SD-R3 compared to cellulose (*q* < 0.05), while *Faecalibacterium* levels were lower in cellulose than in SD-R3 (*q* < 0.05). The *Lachnospiraceae* NK4A136 group was more abundant in SD-FST1.7, SD-R3, and SD-Rye106 compared to cellulose (*q* < 0.05). Notably, *Lactobacillus* was present at significantly higher proportions in all bread groups except for inulin (*q* < 0.05). Furthermore, *Ligilactobacillus* was significantly more abundant in the SD-control and SD-FST1.7 groups compared to cellulose (*q* < 0.05), while *Megamonas* levels increased in SD-FST1.7 relative to cellulose. *Parabacteroides* was reduced in SD-control and SD-R3 groups compared to cellulose (*q* < 0.05). Additionally, *Paraclostridium* levels were significantly lower in SD-control compared to cellulose (*q* < 0.05). *Prevotella_9* showed significantly higher fold changes in SD-FST1.7 compared to cellulose (*q* < 0.05), while SD-R3 and SD-Rye106 also displayed higher abundances close to significance (*q* values nearing 0.05). *Subdoligranulum* levels were elevated in SD-FST1.7 and SD-R3 relative to cellulose (*q* < 0.05), and *Sutterella* was more abundant in SD-control compared to cellulose. Finally, *UCG-002* showed higher abundance in SD-FST1.7, SD-R3, and SD-Rye106 compared to cellulose, while *Veillonella* levels were lower in all groups except inulin (*q* < 0.05).

### 3.4 Short-chain fatty acid analysis

**Figure 5:**
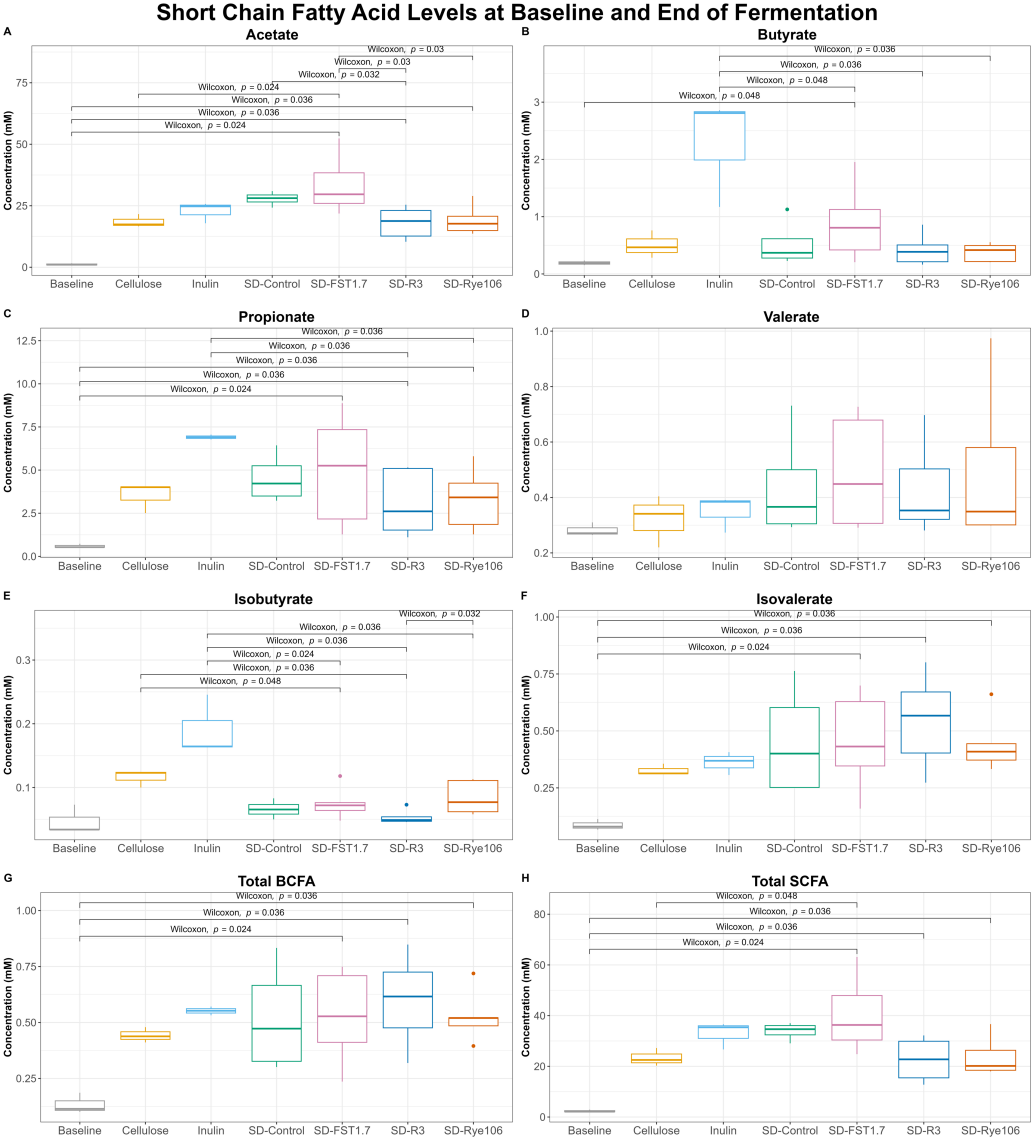
Box plots of short-chain fatty acid (SCFA) concentrations (mM) at baseline (T0) and after fermentation (T12) for cellulose, inulin, SD-Control, SD-R3, SD-Rye106, and SD-FST1.7. **(A)** Acetate, **(B)** butyrate, **(C)** propionate, (E) isobutyrate, **(F)** isovalerate, **(G)** total BCFAs, and **(H)** total SCFAs. Wilcoxon rank test *p* < 0.05.

Short-chain fatty acid analysis revealed a number of significant differences among groups after colonic fermentation (Figure 5). Acetate levels (Figure 5A) were found to be significantly higher in fecal samples fermented with SD-FST1.7, SD-R3 and SD-Rye106 following colonic fermentation compared to baseline (*p* = 0.024, *p* = 0.036, and *p* = 0.036). The concentration of acetate was also found to be significantly higher in fecal samples fermented with SD-FST1.7 compared to samples fermented with cellulose, SD-R3 and SD-Rye106 (*p* = 0.024, *p* = 0.03, and *p* = 0.03) at the end of *in vitro* colonic fermentation. Similarly, following *in vitro* colonic fermentation, fecal samples fermented with SD-Control had higher acetate levels compared to those fermented with SD-R3 (*p* = 0.032). In the presence of SD-FST1.7, fecal samples had significantly higher levels of butyrate at the end of the fermentation compared to baseline (*p* = 0.048) (Figure 5B). Butyrate levels were found to be higher in the fecal samples fermented with inulin compared to those fermented with SD-FST1.7, SD-R3 and SD-Rye106 (*p* = 0.048, *p* = 0.36, and *p* = 0.036).

Fecal fermentation of all substrates including cellulose (negative control) and inulin (positive control) resulted in increased level of propionate (Figure 5C). Comparison of baseline (T0) and T12 revealed significant differences in the samples fermented with SD-FST1.7, SD-R3, and SD-Rye106 (*p* = 0.024, *p* = 0.36, and *p* = 0.036). Fecal samples fermented with inulin displayed significantly higher levels of propionate than samples fermented with SD-R3 and SD-Rye106 (*p* = 0.036 and *p* = 0.036).

Isobutyrate levels (Figure 5E) were significantly higher in fecal samples fermented with cellulose, inulin and SD-Rye106 compared to samples fermented with SD-R3 (*p* = 0.036, *p* = 0.036, and *p* = 0.032). Fecal samples exhibited higher isobutyrate levels at T12 in the presence of inulin, compared to those with SD-FST1.7 and SD-Rye106 (*p* = 0.024 and *p* = 0.036). Similarly, fecal samples fermented with cellulose displayed higher concentration of isobutyrate compared to those fermented with SD-FST1.7 (*p* = 0.048). At the end of colonic fermentation, samples fermented with SD-FST1.7, SD-R3 and SD-Rye106 had significantly higher levels of isovalerate than baseline (*p* = 0.034, *p* = 0.036, and *p* = 0.036) (Figure 5F).

Total branched-chain fatty acid (BCFA) levels (Figure 5G) were found to be significantly higher in samples fermented with SD-FST1.7, SD-R3, and SD-Rye106 compared to baseline (*p* = 0.024, *p* = 0.036, and *p* = 0.036). Similarly, total SCFA levels (Figure 5H) were higher in all samples fermented with SD-FST1.7, SD-R3, and SD-Rye106 at T12 compared to baseline (*p* = 0.024, *p* = 0.036, and *p* = 0.036). Total SCFA levels were significantly higher in fecal samples fermented with SD-FST1.7 than those fermented with cellulose at the end of fermentation (*p* = 0.048).

### 3.5 Correlation between SCFAs and top 25 genera

**Figure 6:**
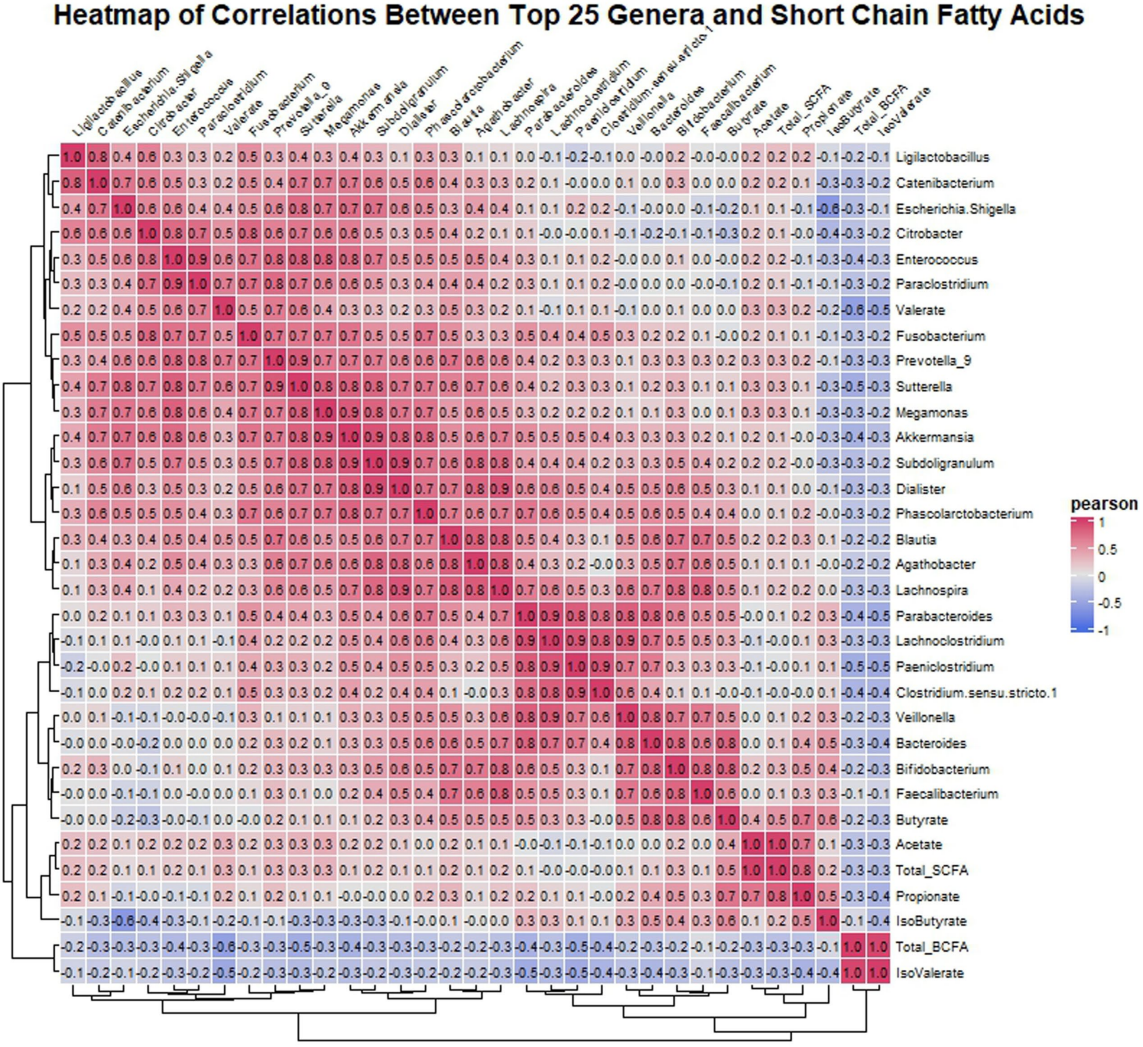
Heatmap displaying Pearson correlation coefficients between the top 25 genera’s relative abundances and SCFA concentrations post-fermentation. Red indicates positive correlations, blue indicates negative, with intensity representing correlation strength.

Pearson correlation analysis was utilized to investigate the relationship between the top 25 genera identified in the microbiota analysis and SCFAs in the gut environment. The analysis revealed several noteworthy correlations (Figure 6). Acetate exhibited a positive correlation with propionate, indicating a potential metabolic relationship between these SCFAs. Moreover, butyrate levels were positively correlated with *Lachnospira*, *Agathobacter*, *Blautia*, *Parabacteroides*, *Veillonella*, *Bacteroides*, *Bifidobacterium*, and *Faecalibacterium*, suggesting a potential role of these genera in butyrate production. Additionally, *Bifidobacterium* showed a positive correlation with propionate, indicating its involvement in propionate metabolism. Similarly, valerate displayed positive correlations with *Citrobacter*, *Enterococcus*, *Paraclostridium*, *Fusobacterium*, *Prevotella*_9, *Sutterella*, and *Blautia*, suggesting potential interactions between these genera and valerate production. In contrast, isovalerate showed negative correlations with all the top 25 genera. Finally, isobutyrate exhibited negative correlations with *Escherichia-Shigella* and *Citrobacter*, while showing positive correlations with *Bacteroides* and *Bifidobacterium*, suggesting differential effects on these genera.

## 4 Discussion

This study provides new insights into how three distinct sourdough breads fortified with selected LAB strains [*L. plantarum* FST1.7 (SD-FST1.7), *L. paracasei* R3 (SD-R3), and *P. pentosaceus* RYE106 (SD-RYE106)] modulate gut microbiome and offer a promising dietary approach for managing symptoms of IBS. Our results unveiled significant alterations in microbial composition and SCFA production following *in vitro* colonic fermentation of various bread formulations, providing a mechanistic understanding of how fortified sourdough breads interact with the gut microbiome to promote gut health. Although a detailed chemical composition analysis post-digestion was not performed in our study, Borowska et al. (2023) provide valuable insights into LAB’s robust utilization of FODMAPs. Their high-throughput FODMAP utilization assay supports our hypothesis that LAB strains, including *L. paracasei* R3 and *P. pentosaceus* RYE106, exhibited robust utilization of fructans, GOS, and other relevant carbohydrates, including fructose and sucrose, which are often implicated in IBS symptomatology. The results revealed that fructan levels decreased by up to 73% with RYE106 and 69% with R3, confirming our hypothesis that the beneficial effects of these sourdough breads are attributed to the substantial reduction of FODMAPs. The observed reductions in maltose, glucose, and fructose after 48 h of fermentation in sourdough bread samples suggest that these strains play a crucial role in mitigating FODMAP-related gut symptoms. Even though our study focuses on the traditional FODMAPs, it is also important to consider microbial-derived carbohydrates such as beta-glucan and dextran, which may have potential effects on IBS symptoms and require further investigation.

While alpha and beta diversity analyses did not reveal significant differences between the bread groups, investigation into relative abundances of taxonomic groups at the phylum and genus levels highlighted significant differences across different bread formulations. Interestingly, Bacteroidota levels were higher in inulin-treated fecal samples compared to those treated with specific sourdough bread formulations, suggesting distinct microbial modulation and highlighting the strain-specific effects of LAB fortification. Increased levels of Firmicutes and Bacteroidota are suggested for healthy gut health (Ponnusamy et al., 2011) and intestinal inflammation is generally associated with reduced Firmicutes and Bacteroidota levels (Ghoshal et al., 2012). Furthermore, commensal species of Firmicutes and Bacteroidota were shown to induce T-regulatory cells and inhibit Th17-mediated inflammation in previous studies (Veiga et al., 2010; Mazmanian et al., 2008; Chow et al., 2010).

At the genus level, several noteworthy changes were observed. *Agathobacter* was elevated in fecal samples fermented with all breads and inulin compared to those fermented with cellulose (ANCOMBC, FDR < 0.05). *Agathobacter* is recognized for its role in butyrate production, which is crucial for maintaining intestinal homeostasis and mucosal integrity (Rios-Covian et al., 2016). The enhanced levels of *Agathobacter* in the presence of SD-R3 and SD-Rye106 breads indicated a synergistic effect between these strains and beneficial gut bacteria, which can facilitate SCFA production and contribute to improved gut health. In contrast, *Bacteroides* levels were lower in fecal samples fermented with SD-FST1.7 and SD-Rye106 compared to those fermented with cellulose, suggesting these specific two LAB strains may be effective in suppressing potentially pathogenic microbial populations associated with IBS (Scott et al., 2014). High levels of *Bacteroides* have been associated with gut dysbiosis and inflammation, while lower levels have been linked to improved gut barrier function and reduced risk of gastrointestinal disorders (Taketani et al., 2020). A recent study reported high levels of *Bacteroides* in severe IBS patients (Tap et al., 2017). Thus, the modulation of *Bacteroides* abundance through dietary interventions such as fortified sourdough breads could represent a novel approach to maintaining a balanced gut microbiota and effectively managing IBS symptoms by targeting dysbiosis. *Limosilactobacillus* (formerly *Lactobacillus*) was the signature genus for samples fermented with SD-FST1.7. *Limosilactobacillus* belongs to *Lactobacillaceae* family and it includes probiotic strains such as *Limosilactobacillus reuteri* (Abuqwider et al., 2022; Ksiezarek et al., 2022). *L. reuteri* has been shown to reduce inflammation in intestine (Tang et al., 2019). Moreover, *Bifidobacterium* levels were the significantly elevated in inulin-treated samples, indicating a potential preference for growth in an inulin-rich environment. *Bifidobacterium*, known for its probiotic properties, plays a crucial role in fermenting dietary fibers and producing beneficial metabolites such as SCFA thereby promoting gut health and alleviating symptoms of IBS (Scott et al., 2014).

The findings also highlighted a reduction in *Veillonella* levels within fecal samples fermented with SD-R3 and SD-Rye106 compared to those fermented with cellulose at the end of *in vitro* colonic fermentation. Elevated *Veillonella* levels have been reported in individuals with IBS and are associated with inflammatory responses (Rigsbee et al., 2012; Tana et al., 2010). These bacteria have lipopolysaccharides which can contribute to gut inflammation and exacerbate IBS symptoms (Rocha et al., 2023). The ability of our sourdough formulations to decrease *Veillonella* offers a novel therapeutic avenue for mitigating IBS symptoms by targeting inflammation at its microbial source, thereby enhancing gut health.

Short-chain fatty acid analysis constituted a crucial aspect of our study, providing new insights into the metabolic interactions within the gut environment and their implications for gastrointestinal health, particularly in individuals with IBS. SCFAs, including acetate, propionate, and butyrate, serve as primary metabolites resulting from the fermentation of dietary fibers by gut microbiota in the colon (Koç et al., 2020; Morrison and Preston, 2016). These SCFAs play multifaceted roles in maintaining gut homeostasis and exerting anti-inflammatory, immunomodulatory, and trophic effects on the colonic epithelium (Morrison and Preston, 2016; den Besten et al., 2013). Significant increases in acetate levels were observed following the fermentation of all sourdough formulations, with notable elevations observed in SD-FST1.7. Acetate, the most abundant SCFA in the colon, has been implicated in promoting gut barrier function, modulating immune responses, and attenuating inflammation (Morrison and Preston, 2016). Furthermore, butyrate, another critical SCFA, demonstrated notable variations across bread samples, with significantly higher levels detected post-fermentation in specific variants, in particular SD-FST1.7. Butyrate serves as a primary energy source for colonic epithelial cells and plays pivotal roles in epithelial integrity, mucosal immune regulation, and inflammation modulation (Rios-Covian et al., 2016). Propionate, the third major SCFA, exhibited intriguing dynamics across fermentation groups, with significant differences observed between baseline and post-fermentation levels in samples fermented with SD-FST1.7, SD-R3 and SD-Rye106. Propionate has garnered attention for its role in regulating appetite, glucose homeostasis, and lipid metabolism, in addition to exerting anti-inflammatory effects within the gut (Chambers et al., 2015). The observed alterations in acetate, butyrate and propionate levels underscore the potential of fortified sourdough breads to modulate metabolic processes and inflammatory pathways, highlighting their implicated in IBS pathophysiology.

The correlation analysis conducted in our study further elucidated the intricate relationship between microbial composition and SCFA production in the gut environment. Positive correlations were observed between specific bacterial genera and SCFA levels, suggesting potential microbial contributors to SCFA production. For instance, butyrate levels were positively correlated with genera known for their butyrate-producing capabilities, such as *Lachnospira*, *Agathobacter*, *Blautia*, *Parabacteroides*, *Veillonella*, *Bacteroides*, *Bifidobacterium*, and *Faecalibacterium* (Rios-Covian et al., 2016). These findings underscore the potential of specific bacterial taxa to modulate SCFA production and contribute to gut health maintenance in individuals with IBS.

In summary, our study offers new insights and mechanistic understanding into how fortified sourdough breads influence SCFA production and microbial composition, highlighting their broader potential in dietary interventions for IBS symptom management. The observed alterations in SCFA profiles and their correlation with specific bacterial genera hold promising implications for the management of IBS symptoms and the promotion of gut health.

While this study utilized an *in vitro* digestion and fermentation model, which is useful for controlled experimentation, it does not fully capture the complexity of the human digestive system; thus, the results may differ in actual human physiology. Additionally, we used pooled fecal samples from healthy individuals to mimic the human gut microbiome. However, individual baseline microbiomes differ, potentially influencing fermentation outcomes in real-world conditions. Future studies should consider *in vivo* experiments or personalized models to validate these findings in clinical settings and explore the long-term effects of fortified sourdough bread consumption on gut microbiota composition and IBS symptom management.

## 5 Conclusion

In conclusion, our study demonstrates the potential of fortified sourdough breads as a dietary intervention for individuals managing symptoms of IBS. By harnessing the fermentative properties of sourdough and incorporating specific LAB strains, we have successfully developed bread variants with reduced FODMAP content and enhanced gut-friendly attributes. Our findings highlight the positive impact of these fortified breads on gut microbiota composition and SCFA production. Among the evaluated bread variants, SD-FST1.7 emerges as a promising candidate, exhibiting favorable outcomes in terms of microbial modulation and SCFA profiles. Moving forward, continued research including human clinical trials will be essential to validate the efficacy and long-term benefits of these gut-friendly bread options in improving the quality of life for individuals managing IBS.

## Data Availability

The raw sequencing data has been updated to NCBI SRA repositories. The accession number of BioProject is PRJNA1168565. https://www.ncbi.nlm.nih.gov/bioproject/PRJNA1168565.
