# Supplementary material for: Impact of low FODMAP sourdough bread on gut microbiota using an in vitro colonic fermentation model
Source: Front Microbiol. 2024 Nov 11;15:1496022. doi: 10.3389/fmicb.2024.1496022 (PMC11586379; doi:10.3389/fmicb.2024.1496022)
Supplement: Supplementary file 1 [file Data_Sheet_1.DOCX]

**Supplementary Method**

**Bioinformatics analysis**

The raw sequencing data underwent processing via the DADA2 pipeline (version 1.16) as outlined by Callahan et al. (1), incorporating specific parameters for optimal data refinement. These parameters included maxN=0, maxEE=c(2, 2), rm.phix=TRUE, truncQ=2, trimLeft=c(17, 21), and truncLen=c(280, 260), ensuring stringent quality control and accurate sequence inference. The processed data were then utilized to generate an amplicon sequence variant (ASV) table and perform taxonomic assignment using the SILVA v138.1 database (2). Integration of sample and group metadata, ASV table, and taxonomic information facilitated the creation of a phyloseq object for comprehensive downstream analyses (3).

Relative abundance was calculated from the phyloseq object using the function:
“transform_sample_counts (phyloseqobject, function (x) {x / sum (x) * 100})”.
Data frames were created from the phyloseq object at the phylum and genus levels for further analysis. The tax_glom() function was used to aggregate taxa at the desired taxonomic rank, while the psmelt() function was employed to convert the phyloseq object to a data frame for visualization. ggplot2 was used to create bar plots from the data frame at the phylum and genus levels.

Alpha diversity metrics, including Observed, Chao1, Shannon, and Simpson indices, were computed to characterize within-sample diversity using the estimate_richness() function. Visualizations were generated using the plot_richness() function on the phyloseq object. To add statistical significance to the plots, the geom_pwc() function from ggpubr was used in conjunction with the Dunn Test, with the p-values adjusted using the false discovery rate (FDR) method.

Beta diversity was assessed using the Bray-Curtis distance matrix and visualized through principal coordinate analysis (PCoA) to examine between-sample dissimilarities. Beta diversity was calculated using the ordinate() function and visualized using the plot_ordination() function. Pairwise PERMANOVA was computed using the pairwise.adonis() function with the parameter p.adjust.m = "fdr" for FDR correction. The microbiome package's transform() function was used to perform centered log-ratio (CLR) transformation on the phyloseq object. A Bray-Curtis distance matrix was created using the phyloseq package's distance() function:
distance(phyloseqobject, method = "bray").

For taxonomic comparisons at the phylum and genus levels, the rstatix package was used to perform Dunn tests. The Dunn test was applied, and FDR was used to correct p-values for multiple comparisons.

To assess differential microbial abundance across the six experimental groups, we employed ANCOM-BC (Analysis of Compositions of Microbiomes with Bias Correction), which accounts for compositional data and applies bias correction to identify significant differences (4). Cellulose (negative control) was used as the reference group for pairwise comparisons. Groups were sorted in the phyloseq object, with "Cellulose" as the reference. The following parameters were used for ANCOM-BC analysis:
p_adj_method = "fdr", formula = "Group", group = "Group", neg_lb = TRUE, tol = 1e-5, max_iter = 100, conserve = TRUE, alpha = 0.05, global = TRUE.

Correlation analysis was performed using the cor_heatmap() function. The top 25 genera were selected for correlation analysis with SCFA levels.

All statistical analyses and graphics were generated using R (version 4.3.2). The following libraries were used: library(dplyr), library(dada2), library(phyloseq), library(tidyr), library(textshape), library(tibble), library(ggplot2), library(ggpubr), library(pairwiseAdonis), library(magrittr), library(tidyverse), library(microshades), library(ggforce), library(ggbiplot), library(pals), library(microbiomeMarker), library(knitr), library(microViz), library(made4), library(ANCOMBC), library(vegan), library(rstatix).
