# Supplementary material for: Impact of low FODMAP sourdough bread on gut microbiota using an in vitro colonic fermentation model
Source: Front Microbiol. 2024 Nov 11;15:1496022. doi: 10.3389/fmicb.2024.1496022 (PMC11586379; doi:10.3389/fmicb.2024.1496022)

**Supplementary Materials**

**Figure S1** Relative abundance of phylum (%) in gut microbiota before and after fermentation
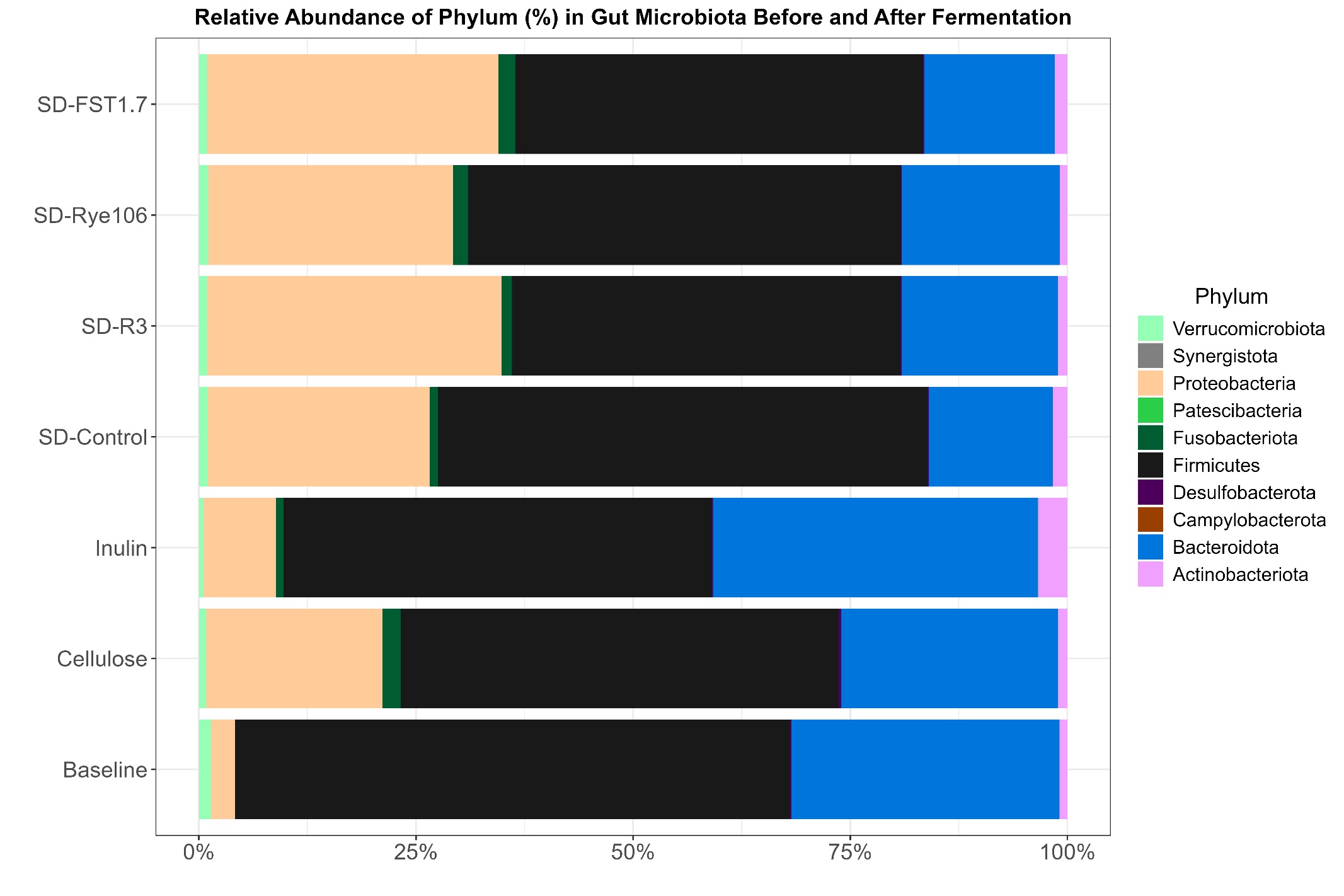
**Figure S2** Relative abundance of top 25 genera (%) in gut microbiota before and after fermentation


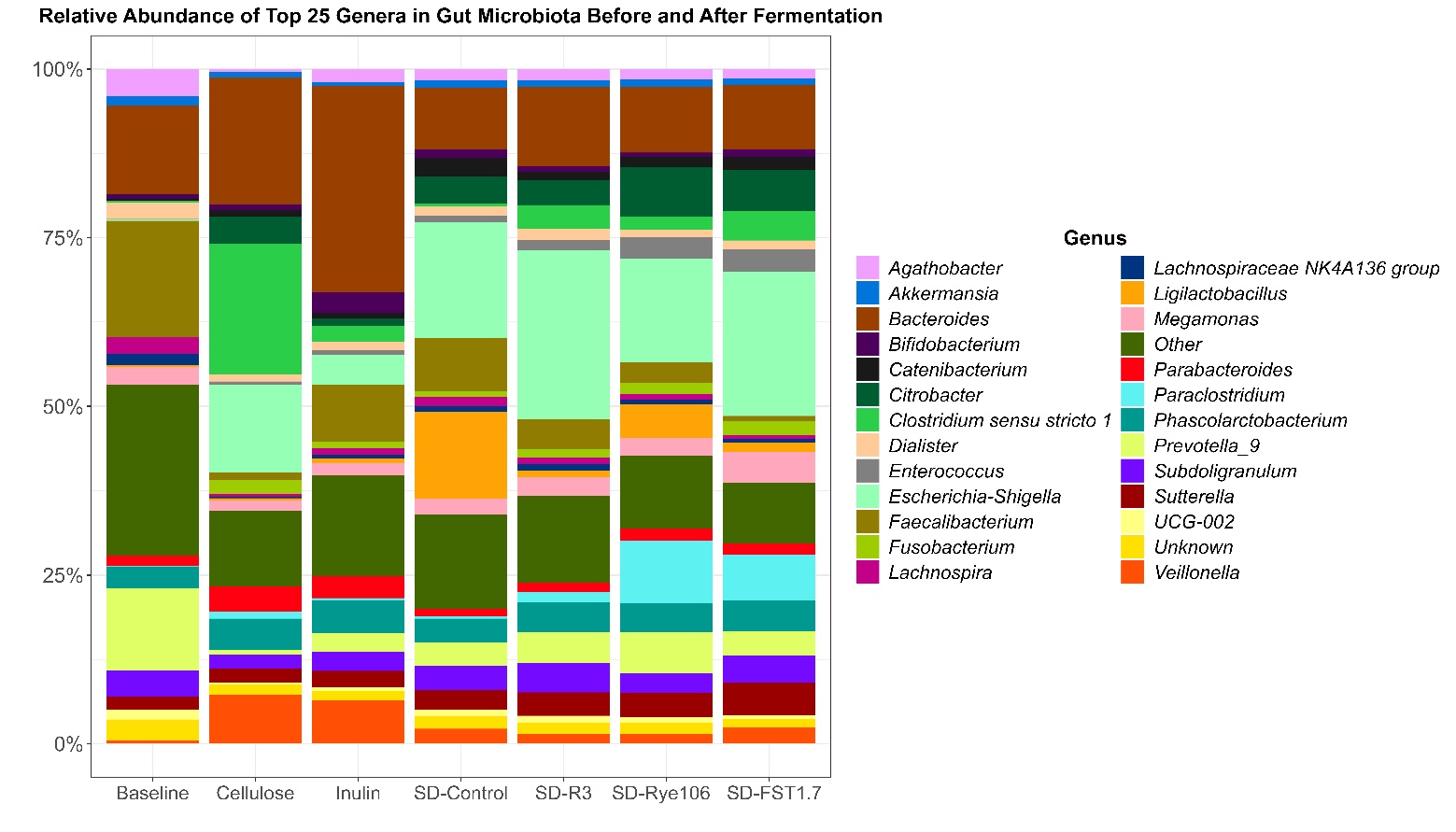

Supplement: Supplementary file 2 [file Data_Sheet_2.DOCX]
